# Electroacupuncture as adjunctive therapy for insomnia via targeting the GABAergic microbiota-gut-brain axis: a mini review

**DOI:** 10.3389/fpsyt.2025.1613408

**Published:** 2025-07-18

**Authors:** Xiao Wang, Lijuan Yan, Xiaodong Zhang, Xiang Liu, Bin Yang

**Affiliations:** ^1^ Department of Ultrasound, The First Affiliated Hospital of Xiamen University, School of Medicine, Xiamen University, Xiamen, China; ^2^ Department of Anesthesiology, The First Affiliated Hospital of Xiamen University, School of Medicine, Xiamen University, Xiamen, China; ^3^ Neuroscience Program, Department of Physiology, Michigan State University, East Lansing, MI, United States; ^4^ Department of Radiology, Michigan State University, East Lansing, MI, United States

**Keywords:** insomnia, electroacupuncture, γ-aminobutyric acid, gut microbiota, microbiota-gut-brain axis

## Abstract

Insomnia, affecting up to 30% of adults (typically 18–65 years), is characterized by GABAergic dysfunction and hyperarousal. This mini-review establishes three pivotal advances in insomnia therapeutics: Firstly, it is demonstrated that microbiota-gut-brain axis (MGBA) dysregulation is mechanistically central to insomnia, directly linking gut dysbiosis to vagal, hypothalamic-pituitary-adrenal (HPA), and γ-aminobutyric acid (GABA) axis dysfunction and neuroinflammation. Secondly, the present study documents the unique multitarget effects of electroacupuncture (EA), which have been shown to simultaneously normalize HPA axis activity, enrich GABA-producing microbiota, improve the vagal tone, and suppress neuroimmune activation. The aforementioned effects collectively resolve insomnia’s multifactorial etiology. Thirdly, clinical evidence confirms the sustained efficacy of EA to be comparable to that of hypnotics, yet with superior safety and durability. EA redefines therapeutic frameworks by integrating biological and neural interventions that are inaccessible to single-target approaches.

## Graphical Abstract

Notes: ↑, upregulating/enhancing. ↓, reducing. EA, electroacupuncture; HPA, hypothalamic-pituitary-adrenal; CHR, corticotropin-releasing hormone; GAB, γ-aminobutyric acid; SCN, suprachiasmatic nucleus.
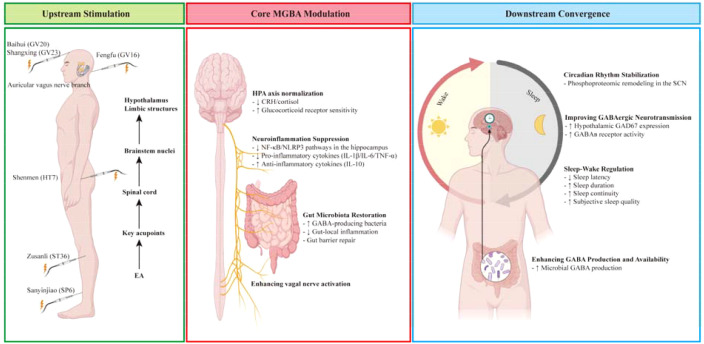

## 1 Introduction

Insomnia is the second most prevalent mental disorder on a global scale (1), affecting up to 30% of adults (typically between the ages of 18 and 65) with severe consequences for public health, occupational functioning, and economic productivity (2–4). Notwithstanding its clinical significance, contemporary therapeutic strategies are encumbered by critical limitations. Hypnotics are associated with the risks of dependency and residual daytime impairment, while cognitive behavioral therapy for insomnia (CBT-I) remains underutilized due to accessibility barriers (1, 5, 6). This unmet need underscores the urgency to elucidate novel pathophysiological mechanisms.

Review evidence primarily from human studies supports the hyperactivation of corticolimbic circuits during both sleep and wake states as a neural substrate of insomnia pathophysiology (7). Concurrently, preclinical research in mice has demonstrated that antibiotic-induced dysbiosis can impact cortical interneuron dendritic morphology (8). Reviews synthesizing this preclinical evidence further suggest that MGBA dysregulation may contribute to insomnia pathophysiology through mechanisms involving GABAergic signaling and HPA axis hyperactivity (9). Neuroimaging studies reveal that insomnia patients exhibit reduced cortical GABA concentrations measured by magnetic resonance spectroscopy (10–12). These observations may be linked to gut microbiota (GM) alterations in chronic insomnia, suggesting GM modulation as a novel therapeutic target to restore GABAergic function in chronic insomnia patients (13). Crucially, gut dysbiosis in these patients depletes GABA-producing bacteria (e.g., *Bacteroides*, *Bifidobacterium*) *(* 14), establishing a bidirectional link between microbial ecology and the central nervous system (CNS) hyperarousal. Recent findings have indicated that insomnia patients exhibit impaired peripheral GABAergic inhibition, which is associated with reduced GABA_A_ receptor α1/α2 mRNA expression. Reduced α1 levels have been shown to predict poorer sleep metrics (sleep quality and sleep time), while diminished α2 has been linked to daytime dysfunction (15). Beyond GABA_A_ receptors, research now implicates GABA_B_ and ρ‐containing GABA_A_ (GABA_C_) receptors in sleep-wake regulation (16).

A recent review suggests traditional Chinese medicine and acupuncture may offer a mechanism-based strategy for treating insomnia guided by the MGBA theory (17). Conventional pharmacotherapies that target specific neurotransmitter systems, electroacupuncture (EA), however, has been shown to modulate the HPA axis, the gut microbiota, and neuroimmune interactions, thereby addressing the multifactorial etiology of insomnia (18–21). This multifaceted regulation of neurochemical, inflammatory, and gut-barrier pathways has the potential to overcome the limitations of current treatments.

## 2 Pathophysiological basis of insomnia: GABAergic dysregulation and gut-brain crosstalk

The etiology of insomnia is multifactorial, with the pathophysiology being driven by gut microbial dysbiosis. The MGBA has been identified as a central regulator, exerting its influence via neuroendocrine (HPA axis), neural (vagal signaling), and neuroimmune (microbial metabolite-mediated) pathways (8, 14, 17, 22, 23). As demonstrated in the extant literature, these pathways collectively disrupt central nervous system function and sleep regulation via GABAergic dysfunction, systemic inflammation, and circadian rhythm disruption (24, 25).

### 2.1 HPA axis dyshomeostasis: a neuroendocrine bridge between stress and sleep disruption

Chronic stress triggers bidirectional gut-brain disturbances involving microbial dysbiosis and intestinal hyperpermeability (26, 27). Based on *in vivo* findings, commensal microbiota calibrates HPA axis responsiveness in mice, as evidenced by amplified stress reactivity in germ-free models compared to specific pathogen-free and gnotobiotic counterparts (28). Several reviews have confirmed that insomnia pathophysiology involves sleep deprivation-induced sympatho-adrenal activation, driving HPA axis hyperactivity characterized by elevated cortisol, corticotropin-releasing hormone (CRH) hypersecretion, and impaired glucocorticoid receptor feedback (27, 29). This initiates a self-perpetuating cycle: stress-induced intestinal permeability facilitates bacterial translocation, activating TLR4/NF-κB pathways that exacerbate systemic inflammation and HPA axis activation (26).

The influence of gut microbes on the HPA axis is a complex process involving the release of neuroactive metabolites, neurotransmitters, and direct neural stimulation. Experimental evidence in gnotobiotic mice demonstrates that *L. rhamnosus* (*JB-1*) modulates HPA axis activity via vagus nerve-mediated pathways, modulating the GABAergic system and reducing stress-induced corticosterone (30). Clinical reviews have identified a further association between gut microbiota-derived neuroactive metabolites and HPA regulation across neurological disorders, highlighting vagal neurotransmission as a conserved mechanism in mammals (31). The influence of short-chain fatty acids (SCFAs) on clock gene expression and sleep patterns suggests a potential role for gut microbiota in propagating the circadian rhythm at the molecular level (32). As demonstrated by preclinical studies, probiotic-treated animals exhibit an attenuated HPA axis response to stress, as indicated by reduced corticosterone elevation (26). In contrast, a double-blind, randomized controlled trial (RCT) in humans undergoing academic stress revealed that *Lactobacillus* casei strain *Shirota* (LcS) maintained sleep quality, suggesting the potential for microbiota-targeted interventions to have clinical relevance (33). As demonstrated in the relevant literature, probiotics have been shown to reverse host metabolic alterations or modulate immune responses associated with gut dysbiosis (*Bifidobacterium* in human irritable bowel syndrome; *Lactobacillus/Bifidobacterium* in maternal separation of rats) (34–36). It is noteworthy that their capacity to modulate corticosterone secretion and reverse HPA axis dysfunction in rodents furnishes a mechanistic framework for investigating the potential of probiotic interventions in the context of insomnia (9, 36).

### 2.2 Vagus nerve mediation in insomnia pathophysiology

The vagus nerve serves as the primary neural conduit for gut-brain communication in insomnia, with 90% of fibers transmitting afferent gut-derived signals—including microbial GABA, serotonin, and norepinephrine—to the CNS (14, 22, 23). The vagal functional mechanism was initiated by the activation and regulation of the HPA axis, which resulted in the generation of CRH. This hormone plays a pivotal role in coordinating the organism’s adaptive stress reaction and maintaining physiological homeostasis (37). Experimental evidence demonstrated that probiotics like *Lactobacillus* and *Bifidobacterium* modulate CNS function via vagus nerve-dependent GABA signaling and systemic neuroactive metabolite circulation (9, 30). Simultaneously, oral *Lactobacillus* administration upregulates GABA receptors in sleep-related brain regions (prefrontal cortex, hypothalamus). This phenomenon is counteracted by vagotomy, as evidenced by the findings of the study (30). Vagal neurotransmission has been demonstrated to enhance emotional regulation through GABAergic inhibition of amygdala CRH neurons, thereby establishing a link between microbial modulation and insomnia-related anxiety (9).

### 2.3 Microbial metabolite crosstalk: neuroimmune axis regulation in insomnia pathogenesis

Gut microbiota orchestrates insomnia through neuroactive metabolite production, immune modulation, and neurotransmitter regulation. The mediation of sleep-wake homeostasis by microbial GABA, serotonin, and SCFAs occurs via bidirectional gut-brain communication (32, 38, 39).

GABA, the predominant inhibitory neurotransmitter, has been demonstrated to modulate thalamocortical synchronization and hypothalamic sleep-promoting circuits (8). Beyond the suppression of neuronal activity, GABA exerts anxiolytic, autonomic-stabilizing, and neuroprotective effects that are critical for the maintenance of sleep. A clinical study has demonstrated that the ingestion of fermented rice germ extract containing GABA has a positive effect on sleep latency and subjective sleep quality in patients suffering from insomnia (40). From a mechanistic perspective, the ingestion of GABA-rich fermented milk has been demonstrated to reduce sleep latency and prolong sleep duration in sub-threshold sodium pentobarbital dose-induced sleep experiments utilizing ICB mice, in comparison to control groups (41). This finding validates conserved physiological pathways. Additionally, GABAergic dysregulation may mediate the bidirectional link between Parkinson’s disease pathogenesis and comorbid sleep disorders (16). In recent neuroimaging studies, the concentrations of glutamate and GABA (in addition to the density and activity of neurotransmitter receptors and transporters) have been associated with mood disorders (13). Neuroimaging reveals insomnia severity correlates with reduced cortical GABA concentrations (measured via magnetic resonance spectroscopy) rather than peripheral levels (16, 41, 42), emphasizing central GABAergic tone’s clinical relevance.

Dysbiosis-induced GABA depletion has been demonstrated to trigger glutamatergic excitotoxicity and neuroinflammation, which are recognized as key drivers of sleep-wake dysregulation (3, 43). The regulation of GABA metabolism by gut microbiota is achieved through the synthesis of GABA by *Lactobacillus* and *Bifidobacterium* via glutamate decarboxylase (14, 44) and GABA utilization by *Bacteroides* and *Parabacteroides* as a nitrogen source, creating bidirectional host-microbe metabolic crosstalk (45). GABA receptor agonists and uptake inhibitors have been demonstrated to effectively regulate sleep (16, 46), while GABA supplementation is promising for both sleep initiation and maintenance (40, 41, 46, 47). It is hypothesized that these effects may involve interactions with SCFAs, such as butyrate, which are critical gut-brain mediators (38, 41, 48). While exogenous GABA’s BBB permeability remains debated, it exerts indirect neural effects via gut pathways, including modulating microbiota and stimulating GABA_B_ receptors expressing on intestinal/vagal afferents (41). Given these multifaceted interactions, therapeutic strategies targeting GABAergic-microbiome interactions hold promise for insomnia treatment (39), warranting further mechanistic studies

## 3 EA’s possible multimodal regulation of GABAergic MGBA in insomnia and coexisting diseases therapeutics

EA modulates the MGBA to address central GABAergic dysfunction and gut dysbiosis (18, 19, 48, 49), thereby demonstrating superior anti-insomnia effects compared to conventional therapies.

HPA Axis Modulation: The anti-insomnia effects of EA appear particularly robust in regulating HPA axis hyperactivity, a well-established feature of insomnia pathophysiology (50). By modulating HPA axis hyperactivity, enhancing vagal tone, and restoring microbial production of sleep-regulating metabolites, EA targets the multifactorial etiology of insomnia. In a mouse model of depression, EA synergizes electrical stimulation with acupuncture to uniquely regulate central GABAergic neurotransmission and gut microbial ecology, specifically modulating the abundance of *Lactobacillus* and *staphylococci (* 19). In SPF Sprague-Dawley rats under cage-change-induced insomnia, EA at Ganshu (BL18) and Zusanli (ST36) reduced wakefulness and increased non-rapid eye movement (NREM) sleep. Mechanistically, EA regulated hypothalamic dopamine (DA) and DA receptors (D1R/D2R) within the HPA axis, counteracting stress-induced neurotransmitter alterations to normalize sleep-wake cycles (50). In addition, in the context of the maternal separation rats model, the efficacy of acupuncture in reducing corticosterone (CORT) and ACTH levels in plasma, as well as the hypothalamic immunoreactivity (IR) of arginine vasopressin (AVP) in the hypothalamic paraventricular nucleus, has been well-documented (51, 52).

Gut Microbial Regulation: Under physiological and pathological conditions, gut microorganisms can influence the functions and behaviors of the brain through bidirectional regulation of various immune, endocrine, and vagus nerve pathways via the gut-brain axis (53). EA inhibits peripheral inflammation by balancing gut microbiota, the attenuating hippocampal neuroinflammation (54) (**Table 1**). In chronic restraint stress-induced anxiety disorders mouse, EA at Baihui (GV20) partially alleviated anxiety-like behavior and mitigated gut microbiome dysbiosis (55). In C57BL/6 male mice with p-chlorophenylalanine (PCPA)-induced insomnia, acupuncture at Baihui (GV20), Sanyinjiao (SP6), and Shenmen (HT7) ameliorated sleep disturbances through regulating the gut flora to modulate the host immune response (56). In addition, longitudinal metagenomic analyses demonstrate that a standardized 8-week EA regimen induces significant ecological shifts in D-galactose-induced Alzheimer’s disease (AD) model rats, marked by significant enrichment of GABA-producing *Lactobacillus* and *Bifidobacterium* alongside depletion of *Streptococcus* and *Enterococcus (* 57). These microbial changes have been shown to correlate with measurable neurochemical alterations, including elevated GABA and glutamate levels in the hypothalamus and peripheral blood (58). It is noteworthy that EA has been observed to alleviate symptoms of depression-like behaviors by regulating *Lactobacillaceae* and *Bacteroidaceae (* 59, 60), a finding that aligns with the insomnia model. However, while these mechanisms have been established in models of central nervous system disorders, rigorous validation in insomnia-specific paradigms remains essential.

**Table 1:** Preclinical studies included in this mini review.

Refs.	Models	Acupoints	GABA	Inflammatory markers	Substances involving sleep-wake	Gut-microbiota composition
Hong J et al (56)	PCPA-induced insomnia mice	GV20, SP6, HT7	–	–	↓ DA, 5-HT and NE	↑ *Firmicutes*/Bacteroidetes ratio ↑ *Lactobacillus* ↓ *Clostridium XlVb, Lachnospiracea incertae sedis, Anaerovorax, Oscillibacter, Pseudoflavonifractor*, and *Acetatifactor*
Zhang F et al (58)	PCPA-induced insomnia mice	Cymba concha	↑ GABA in the hypothalamus and peripheral blood	–	–	–
Li G et al (66)	multiple-platform apparatus-induced sleep disorder mice	GV20	–	↓ IL-1β, MCP-1 and TNF-α in the hippocamp ↑ IL-10 in the hippocamp	↓ TLR4/NF-κB	–
Qiu X et al (19)	CUMS-induced depression mice	GV20, GV29	–	–	–	↑ *Lactobacillus* ↓ *staphylococci*
Cai W et al (59)	CUMS-induced Poststroke depression rats	GV20, GV24	–	–	–	↑ *Lactobacillaceae* and *Bacteroidaceae*
Li P et al (60)	CUMS-induced depression rats	GV23, PC7	–	–	↓ DA and 5-HT in serum and hippocampus	↓ *Bacteroidetes/Firmicutes* ratio
Li J et al (64)	CUMS-induced depression rats	GV16, GV23	↑ GABA_B_ in the lateral habenula nucleus	↓ IL-1β and IL-6 in the lateral habenular nucleus ↑ IL-10 levels in the lateral habenular nucleus	↓ NF-κB/NLRP3 in the lateral habenular nucleus ↑ DA、5-HT、NE in the lateral habenular and serum.	–
Bai J et al (55)	Chronic restraint stress-induced anxiety disorders mouse	GV20, the tail	–	–	–	↑ Phylum *Candidatus_Melainabacteria;* ↑ Family *Prevotellaceae* and *unclassified_o_Vampirovibrionales;* ↑ Genus *Faecalimonas, Vampirovibrio* and *Lachnoclostridium;* ↑ Species *Faecalimonas_umbilicata, Vampirovibrio_chlorellavorus* and *unclassified_g_Lachnoclostridium*
Jiang J et al (54)	Alzheimer’s disease models using SAMP8 mice	GV20, GV29	–	↓ IL-1β, IL-6 and TNF-α in serum and hippocampus	–	↑ *Bacteroidia* and *Clostridia*
Xiao M et al (57)	D-galactose-induced Alzheimer’s disease rats	ST36, GV20	–	–	↓ 5-HT in the colon and hippocamp	↑ *Lactobacillus* and *Bifidobacterium* ↓ *Streptococcus* and *Enterococcus*
Tang L et al (68)	Isoflurane-induced PND mice	GV20, PC6, LI4	–	↓ IL-β、IL-6 and TNF-α in the hippocamp		↑ Lactobacillaceae
Vega-Garcia A et al (62)	Kainic acid-induced status epilepticus rats	DM26	↑ GABA	–	–	–

Notes: ↑, upregulated by EA/acupuncture; ↓, downregulated by EA/acupuncture. GABA, γ-aminobutyric acid; PCPA, p-chlorophenylalanine; CUMS, chronic unpredictable mild stress; PND, perioperative neurocognitive disorders.

Neural Mechanisms: The neural mechanisms underlying EA’s effects involve multiple complementary pathways that enhance central GABAergic tone. The modulation of sleep patterns is contingent upon the activity of discrete populations of GABAergic neurons. It has been demonstrated that elevations in GABA levels facilitate both sleep initiation and maintenance (61). This is achieved through EA-induced stimulation of the auricular vagus nerve branch of PCPA-induced insomnia models in mice, which increases hypothalamic and peripheral blood GABA concentrations (58). The hypothesis that EA stimulates hypothalamic GABAergic neurons has been posited. These neurons have been shown to inhibit hyperactive neural circuits in the limbic system and prefrontal cortex. Presynaptically, in status epilepticus models induced by kainic acid in Sprague-Dawley rats, EA has been shown to enhance GABA synthesis capacity by upregulating glutamic acid decarboxylase (GAD67) expression (62). Notably, by modulating the release of neurotransmitters like serotonin (5-HT), DA, and norepinephrine (NE), GABA_B_ receptors thereby influence essential neural mechanisms encompassing synaptic transmission, plasticity, precursor cell proliferation, and survival pathways in neurons (63). Postsynaptically, in models of chronic unpredictable mild stress-induced depression in Sprague-Dawley rats, EA at Shangxing (GV23) and Fengfu (GV16) elevated GABA_B_ receptor expression, promoting synaptic plasticity while suppressing NF-κB/NLRP3-driven neuroinflammation (64). In addition, EA exerts its neuroprotective effects primarily by suppressing inflammation in the hippocampus. IL-1β has been demonstrated to potentiate GABAergic neurotransmission in a bidirectional manner, with the capacity to enhance presynaptic GABA release in preoptic/anterior hypothalamic neurons and to amplify postsynaptic GABA responses across a range of experimental models (65).

Cytokines or Inflammatory Markers: EA has been demonstrated to attenuate sleep deprivation-induced upregulation of pro-inflammatory cytokines (IL-1β, MCP-1, TNF-α) while elevating anti-inflammatory IL-10 expression (66). Similarly, in models of acute colitis, EA intervention has been observed to induce parallel cytokine modulation in plasma (67). Concurrently, EA reduces circulating pro-inflammatory cytokines (such as IL-6 and TNF-α), creating an optimal microenvironment for GABAergic neurotransmission (68, 69). Integrated cytokine or inflammatory markers effects converge on wakefulness modulation through three principal aspects: spatially via vigilance-regulating brain regions (hypothalamus, basal forebrain, brainstem) where IL-1β alters neuronal discharge and IL-1β/TNF-α exhibit diurnal rhythms (65, 70–72); neurally through vagus nerve signaling where peripheral IL-1β binds paraganglia receptors projecting to brainstem solitary nucleus (73); and molecularly via biochemical cascades involving adenosine/NF-κB/PGD_2_, neurotransmitters (GABA/glutamate/NE), and somnogenic hormones (GHRH/CRH) (65). It is hypothesized that pro-inflammatory cytokines may also exert some of their sleep-modulating effects via GHRH (65).

Circadian Rhythm: It has been demonstrated that glutamatergic and GABAergic synapses exhibit significant molecular enrichment with regard to the regulation of the sleep-wake cycle. The regulation of cortical arousal and wakefulness is primarily governed by dual neurochemical systems. These systems consist of brainstem monoaminergic cell groups (noradrenergic cells, serotonergic cells, histaminergic neurons, and dopaminergic neurons) and basal forebrain (BF) neurons. The latter are predominantly cholinergic and GABAergic subtypes (65). The basal forebrain functions as a pivotal relay station where hypocretin (Hcrt) neurons from the lateral hypothalamus regulate arousal states (74), and the absence of Hcrt neurotransmission leads to frequent transitions between wakeful and sleep states (75). Acupuncture modulates sleep-wake regulation through convergent main areas of the brain’s structures as well as balancing wake-promoting neurotransmitters (NE, serotonin, histamine, dopamine, acetylcholine) and sleep-promoting substances (GABA, opioids) through comprehensive coordination of multiple targets, levels, links, and pathways (76, 77). Furthermore, the most significant enriched phosphor-proteins and phosphosites are involved in post-synapse and glutamatergic synapses. EA has been demonstrated to induce circadian resynchronization through phosphoproteomic remodeling in the suprachiasmatic nucleus (SCN), with phosphorylation events serving as the primary regulatory mechanism. The sleep-wake cycle is subject to modulation by glutamatergic and GABAergic synaptic pathways, which adjust the levels of glutamate and GABA within the SCN (78).

Clinical Evidence: Randomized controlled trials (RCTs) provide compelling clinical evidence for these mechanisms (**Table 2**). In a multicenter RCT, EA significantly reduced Insomnia Severity Index (ISI) scores compared to sham-EA and usual care, with effects sustained at 8-week and 12-week follow-ups (79). In a multicenter study of 270 patients with comorbid insomnia and depression, EA with standard care outperforms sham acupuncture with standard care and standard care alone in sleep quality (80). In a double-dummy, single-blinded RCT involving patients with primary insomnia, six-week acupuncture treatment demonstrated significantly greater improvements in sleep quality, total sleep time, sleep efficiency, and daytime functioning compared to sham acupuncture, effectively facilitating the reestablishment of normal sleep-wake cycles (81). In a single-blind RCT, patients diagnosed with chronic insomnia were administered acupuncture at Baihui (GV20), Yintang (GV29), bilateral Shenmen (HT7), and bilateral Sanyinjiao (SP6). The intervention was administered thrice weekly (every other day) for four weeks, and it was found to have a significant effect on the quality, efficiency, and latency of sleep (82). In addition, a systematic review and meta-analysis also demonstrated EA’s efficacy in managing cancer-related insomnia, evidenced by significant increases in total sleep duration and reductions in sleep disruptions (83). Although multicenter RCTs validate EA’s clinical efficacy, reproducibility remains limited by heterogeneous stimulation parameters and non-standardized acupoint selection protocols varied across studies.

**Table 2:** Clinical studies included in this mini review.

Refs.	Frequency and duration	Severity of disease	Age	Acupoint selection
Lee B et al (79)	A 30-minute treatment, 10 sessions (2–3 times a week for 4 weeks)	Patients with insomnia Difficulty in initiating or maintaining sleep, or early awakening, occurring at least three times per week for a period of three months or more. ISI score of at least 15.	Aged 19 to 64 years	GV20, EX-HN3, bilateral HT7, PC6, BL63, and KI4
Yin X et al (80)	A 30-minute treatment, 3 times per week (once every other day except Sunday) for 8 weeks	Patients with insomnia and depression diagnosed from Diagnostic and Statistical Manual of Mental Disorders (Fifth Edition) criteria for depression PSQI was greater than 7 HDRS-17 score of 20 to 35	Aged 18 to 70 years	GV20, GV24, GV29, EX-HN22, HT7, PC6, and SP6
Guo J et al (81)	A 30-minute treatment, 1 time every other day for six weeks	Patients with insomnia diagnosed from DSM-IV-TR (Fourth Edition) Experienced insomnia for 4 weeks or longer	Aged 25 to 75 years	DU24, EX-HN1, DU20, SP-6, and HT-7
Liu C et al (82)	A 30-minute treatment, 3 times a week (once every other day) for 4 weeks	Patients with insomnia diagnosed from the ICSD-3 PSQI score >5 points HAMA score ≥7 points HAMD score ≥14 points	Aged 18 to 70 years	GV20, GV29, HT7 (bilateral), and SP6 (bilateral)

SI, Insomnia Severity Index; PSQI, Pittsburgh Sleep Quality Index; HDRS-17, 17-item Hamilton Depression Rating Scale; HAMA, Hamilton Anxiety Rating Scale; HAMD, Hamilton Depression Rating Scale.

## 4 Future perspectives

It is recommended that subsequent studies examine EA’s parameters in greater detail, with particular attention to stimulation intensity, frequency, duration, and repetition rate, to optimize intervention efficacy. Multi-omics integration (metagenomic/metabolomic/proteomic) to decode strain-specific microbial-GABA interactions and their neurocircuitry impacts should be considered, particularly in aging populations with metabolic dysfunction. Our recently published study protocol in *Frontiers in Neurology (* 84) has provided a methodological foundation for such studies. Finally, it is necessary to explore synergistic interventions combining EA with probiotics to potentiate MGBA modulation and amplify therapeutic outcomes.

## 5 Conclusion

EA presents a transformative non-pharmacological intervention for insomnia management, offering superior safety through multilevel MGBA modulation. This modulation simultaneously normalizes HPA axis activity, enriches GABA-producing microbiota, enhances vagal tone, and suppresses neuroinflammation.
